# Inhibitory Molecules PD-1, CD73 and CD39 Are Expressed by CD8^+^ T Cells in a Tissue-Dependent Manner and Can Inhibit T Cell Responses to Stimulation

**DOI:** 10.3389/fimmu.2021.704862

**Published:** 2021-07-15

**Authors:** Corinne J. Smith, Christopher M. Snyder

**Affiliations:** Department of Microbiology and Immunology, Sidney Kimmel Medical College, Sidney Kimmel Cancer Center, Thomas Jefferson University, Philadelphia, PA, United States

**Keywords:** CD8 T cells, tissue-resident CD8^+^ T cell, PD-1, CD73, cytomegalovirus (CMV)

## Abstract

The salivary gland is an important tissue for persistence and transmission of multiple viruses. Previous work showed that salivary gland tissue-resident CD8^+^ T cells elicited by viruses were poorly functional *ex vivo*. Using a model of persistent murine cytomegalovirus (MCMV) infection, we now show that CD8^+^ T cells in the salivary gland and other non-lymphoid tissues of mice express multiple molecules associated with T cell exhaustion including PD-1, CD73 and CD39. Strikingly however, these molecules were expressed independently of virus or antigen. Rather, PD-1-expressing T cells remained PD-1^+^ after migration into tissues regardless of infection, while CD73 was activated on CD8^+^ T cells by TGF-β signaling. Blockade of PD-L1, but not CD73, improved cytokine production by salivary gland T cells *ex vivo* and increased the expression of granzyme B after stimulation within the salivary gland. Nevertheless, salivary-gland localized CD8^+^ T cells could kill PD-L1-expressing targets *in vivo*, albeit with modest efficiency, and this was not improved by PD-L1 blockade. Moreover, the impact of PD-L1 blockade on granzyme B expression waned with time. In contrast, the function of kidney-localized T cells was improved by CD73 blockade, but was unaffected by PD-L1 blockade. These data show that tissue localization *per se* is associated with expression of inhibitory molecules that can impact T cell function, but that the functional impact of this expression is context- and tissue-dependent.

## Introduction

Multiple viruses infect the salivary gland and are shed into saliva for transmission to new hosts, including examples such as cytomegalovirus, Epstein Barr Virus, Human Herpesvirus 6, mumps virus, rabies virus, and even SARS-CoV-2 (1–7). Thus, it is important to understand anti-viral T cell behavior in the salivary gland and other sites of viral shedding.

Many labs, including our own, have used the murine cytomegalovirus (MCMV) model to study viral infection of the salivary gland and the resulting T cell responses (8–16). Cytomegalovirus is a member of the beta-subfamily of herpesviruses that infects the majority of people world-wide. Primary infection typically occurs through contact with infected secretions (e.g. saliva, urine or breast milk), after which the virus spreads systemically in the body of the new host (17). CMV establishes latency in many cells throughout the body and is never eradicated but is thought to undergo regular attempts to reactivate and spread (18). Thus, the immune system, and T cells in particular, must maintain continuous vigilance in order to control CMV for the life of the host.

The prolonged virus/host détente leads to profound changes to the T cell compartment of the host. In humans and mice, CMV-specific CD8^+^ T cells in circulation are sustained at high frequencies in an antigen-dependent manner, or even accumulate over time in a process that has been called “memory inflation” (18–24). Evidence suggests that these T cells in mice and humans suppress viral gene expression and control viral reactivation and viral spread (9, 25, 26). Remarkably, while the ongoing stimulation causes marked differentiation of CMV-specific T cells, the circulating T cells remain functional effector cells that can suppress virus and do not exhibit the phenotype, gene signature or functional defects associated with T cell exhaustion including loss of cytokine production and cytolytic capacity, and upregulation of immune checkpoint molecules such as PD-1 (22, 26–31).

In contrast to most tissues in the body, HCMV replicates persistently in sites of shedding including the salivary gland and kidney, and is known to be shed in saliva and urine for prolonged periods of time in humans (4, 32). Similarly, MCMV persistence has been shown in the salivary glands and kidneys of mice (33, 34), although the salivary gland is commonly considered to be the primary site of viral persistence in mice while shedding urine has been much less studied and may be restricted to perinatal infections (35). It is still unclear whether T cells in these sites of viral persistence and shedding remain functional. Foundational studies from Jonjic and colleagues demonstrated that MCMV-specific CD8^+^ T cells were unable to control MCMV in the salivary gland (8, 36). Subsequent work showed that MCMV-specific CD8^+^ T cells migrated into the salivary gland where they largely developed a tissue-resident memory (T_RM_) phenotype (i.e. CD103^+^, CD69^+^) in a TGF-β-dependent manner (13, 14). Viral immune evasion mechanisms clearly support viral titers and persistence in the salivary gland by removing MHC-I from infected epithelial cells (11, 37), implying that T cells are able to affect viral growth. Moreover, CD8^+^ T cells can control viral reactivation from latency in the salivary gland (9). However, T cells in the salivary gland have been shown to be poor at producing cytokines *ex vivo* after restimulation in both MCMV and lymphocytic choriomeningitis virus (LCMV) models (14, 38).

Because of the persistent replication of MCMV in the salivary gland, and the apparent functional defects of T cells recovered from the salivary gland, we evaluated T cells in this tissue for markers of T cell exhaustion. We found that MCMV-specific T cells in multiple tissues, including the salivary gland and kidney expressed PD-1, CD39 and CD73 at steady state, markers that are associated with recent antigen stimulation and T cell exhaustion. Remarkably, expression of these molecules was independent of antigen and MCMV, but instead associated with tissue localization. Blockade of the PD-L1, but not CD73, improved the function of salivary gland-localized T cells. In contrast, cells in the kidney were unaffected by PD-L1 blockade, but displayed improved function after CD73 blockade. Thus, tissue localization *per se* is associated with expression of inhibitory molecules, but the functional impact of this expression is context-dependent.

## Materials and Methods

### Mice

In most cases, mice were originally purchased from Jackson labs and bred in house. C57BL/6J (B6) mice were used for direct infections adoptive transfer recipients. OT-Is on a B6 background (C57BL/6-Tg(TcraTcrb)1100Mjb/J) were bred to congenic CD45.1 expressing mice (B6.SJL-*PtprcaPepcb*/BoyJ). For some experiments, CD45.1^+^ OT-Is were bred onto a RAG^-/-^ (B6.129S7-*Rag1^tm1Mom^*/J) background. OT-I mice deficient in the TGF-β-RII were kindly provided by Dr. Nu Zhang (UT Health, San Antonio) and generated by breeding floxed TGFβRII mice (B6;129-*Tgfbr2^tm1Karl^*/J) to mice expressing the Cre recombinase from the distal LCK promoter (B6.Cg-Tg(Lck-icre)3779Nik/J). By crossing these mice to CD45.1^+^ OT-Is and intercrossing, we ultimately generated OT-I littermates that differed at the CD45 locus and were either wild-type or TGF-β-RII^-/-^. Splenocytes from CD45.2^+^ TGF-βRII^-/-^ OT-Is were mixed with splenocytes from wildtype OT-I littermates that expressed both CD45.1 and CD45.2. The mixed splenocytes were then transferred into CD45.1^+^ recipients. All experiments were approved by the Institutional Animal Care and Use Committee at Thomas Jefferson University.

### Viruses and Infections

Viruses were grown and titered as described previously (39). Mice were infected by the intraperitoneal (i.p.) route with 2x10^5^ pfu of either K181-tfr-OVA, expressing a membrane bound version of full-length OVA (40), and called MCMV-OVA here, or K181 (wild type MCMV) as indicated in the figure legends. Mice were considered chronically infected after 12 weeks. Viral replication in the organs was determined by plaque assay (39, 41). To determine viral load in the organs, genomic DNA was isolated with the Puregene Core A kit (Qiagen) and qPCR for the E1 gene was performed as described previously (42, 43).

### Adoptive Transfers

To seed naïve mice with OT-Is, 1000 splenocytes from congenic OT-Is were transferred retro-orbitally into recipient mice one day prior to infection. For *in vitro* activated OT-Is, splenocytes from OT-I mice were cultured in T cell media (RPMI with 1% Pen/strep, 10% FBS and 0.05 mM β-mercaptoethanol) at a concentration of 4x10^6^ cells/ml with 1 μg/ml of the SIINFEKL peptide. On day 3 of culture, cells were washed and either injected into naïve recipient mice *via* the retro-orbital sinus, or washed and cultured in T cell media for 3 additional days with 30 U/ml of IL-2, but with no peptide to generate PD-1^low^ cells for transfer into naïve recipient mice.

### Antibody Blockades

For the PD-L1 blockades, mice were injected i.p. with 200 μg of anti-PD-L1 (clone 10F.9.G2) or isotype control (clone LTF-2) in 100 μL of PBS every two days for a period of two weeks unless otherwise indicated. For CD73 blockades, mice were injected i.p. with 200 μg of anti-CD73 (clone TY/23) or isotype control (clone 2A3) every other day for two weeks. All antibodies for *in vivo* blockades were purchased from Bio-X-Cell.

### Isolation of Lymphocytes and FACS Staining

Lymphocytes were isolated from the spleen and non-lymphoid organs as described previously (23). In brief, salivary gland and kidney homogenates were suspended in 40% Percoll and layered over on top of a 55% and 75% layered Percoll gradient. Samples were spun for 25 minutes at 600 x g and cells were collected from the interface between the 55% and 75% layers. Staining for FACS was done as before (13, 23), with the addition of antibodies against PD-1 (clone 29F.1A12), 2B4 (clone m2B4(B6)458.1), Lag3 (C9B7W), TIM-3 (B8.2.C12), CTLA-4 (clone UC10-4B9), CD73 (clone TY/11.8) and CD39 (clone Duha59), CD160 (clone 7H1), IFN-γ (clone XMG1.2), TNF-α (clone MP6-XT22), CD107a (clone 1D4B), PD-L1 (clone 10F.9G2), E-cadherin (DECMA-1), Granzyme B (clone QA16A02), Ki67 (B56), pAKT (SDRNR), pS6 (cupk43k). All antibodies were purchased from Biolegend or BD Biosciences. All intracellular stains, including cytokines and phospho-proteins, were performed after permeabilization with the BD Fix/Perm Kit (BD Biosciences). For Ki-67, the BD Permeabilization Buffer Plus was used for the nuclear permeabilization after the initial fixation/permeabilization. Tetramers loaded with B8R peptide were provided by the National Institutes of Health Tetramer Core Facility (http://tetramer.yerkes.emory.edu/) and used as described (22). In all experiments, i.v. antibody labelling was used to distinguish between vascular and parenchymal localized CD8^+^ T cells as described previously (23). Throughout, data from non-lymphoid organs show cells unlabeled by the i.v. antibody, indicating their presence outside of the vasculature of the organ. Splenic data include all T cells, regardless of i.v. antibody labeling. Flow cytometry data was collected on a Fortessa (BD Biosciences) and analyzed using FlowJo version 10 (Treestar). Histograms are shown as modal plots for comparison.

### *In Vitro* Intracellular Cytokine Staining

*In vitro* stimulation for intracellular cytokine staining was done as described previously (44) with minor modifications. Briefly, 5x10^5^ DC2.4s (kindly provided by Dr. Aron Lukacher) were plated into each well of a flat-bottom 96 well plate. 4 hours after plating, cells were treated with 100 ng/ml of IFN-γ to upregulate PD-L1. The following day, DC2.4 cells were pulsed with 10 μg/ml of peptide for 5-6 hours and were washed 3 times before T cells were added. When blocking antibodies were used, they were used at a final concentration of 60 μg/ml and were added to the DC2.4 cells 30 minutes before the addition of T cells. T cells from salivary gland and kidneys were isolated as described above. Spleen homogenates were enriched for T cells by negative selection with biotin labelled antibodies against CD4, CD19, Ter-119 and MHCII and using MojoSort Streptavadin Nanobeads (Biolegend). In all cases, T cells were added to peptide pulsed DC2.4s and incubated for 5 hours at 37 degrees C in the presence of brefeldin A and were subsequently stained for intracellular cytokines and phospho-proteins using the BD Fix/Perm kit using the manufacturers recommended protocol.

### *In Vivo* Intracellular Cytokine Staining (ICS)

An *in vivo* intracellular cytokine assay was based on a previously published protocol (38). In brief, mice were injected i.v. with 200 μg of SIINFEKL and another 50 μg of SIINFEKL was injected directly into the salivary gland. For intraglandular injections, mice were anesthetized with isoflurane using a nose cone and the salivary gland was located by palpating the neck area. A total of 50 μl of solution was injected into the salivary gland with roughly half injected into each lobe. Mice were sacrificed 6 hours after injection. Organs were harvested and processed in media containing 1 μg/ml of brefeldin A and stained for intracellular cytokines as normal. In some experiments, mice were injected i.v. with CFSE simultaneously with peptide as described previously (13).

### *In Vivo* Killing Assay

For target cells, splenocytes were harvested from naïve mice and resuspended at 10^7^ cells/ml. Cells were pulsed with 1 μg/ml of either the SIINFEKL or an irrelevant peptide (B8R) at 37 degrees for one hour. Cells pulsed with the SIINFEKL peptide were labelled with 1 μM Cell trace far red while cells pulsed with the B8R peptide were labelled with 5 μM CFSE. Peptide pulsed populations were mixed and injected i.v. and i.g. into mice. Mice were sacrificed after 48 hours and half of each organ was used to isolate lymphocytes for FACS analysis. The remaining half of each organ was frozen in OCT media for histological sectioning. Sections were prepared and stained with an antibody against E-cadherin (clone DECMA-1) and DAPI as described previously (13). Images were captured on a Nikon Eclipse E800 with NIS Elements Imaging Software and analyzed with Image J (45).

### Statistical Analyses

Throughout data are shown as a mean of a group. In most cases, data were pooled from multiple independent experiments and combined for the graph. In these cases, error bars indicate the standard error of the mean. In some cases, a representative experiment consisting of multiple mice is shown if, for example, batch effects were apparent or different protocols were used. In these cases, error bars indicate the standard deviation of the group. Animal numbers, whether the data were pooled from multiple experiments, and the number of independent replicate experiments are indicated in the figure legends. Statistical significance was assessed with pairwise comparisons between 2 groups using either paired or unpaired two-tailed students’ t-tests, or a one sample t-test, as appropriate and indicated in the figure legends. In all cases, asterisks indicate p-value thresholds as follows: *p ≤0.05, **p≤0.01, ***p≤0.001, ****p≤0.0001.

## Results

### Multiple Inhibitory Molecules Expressed on MCMV Specific Tissue-Resident T Cells

**Figure 1:**
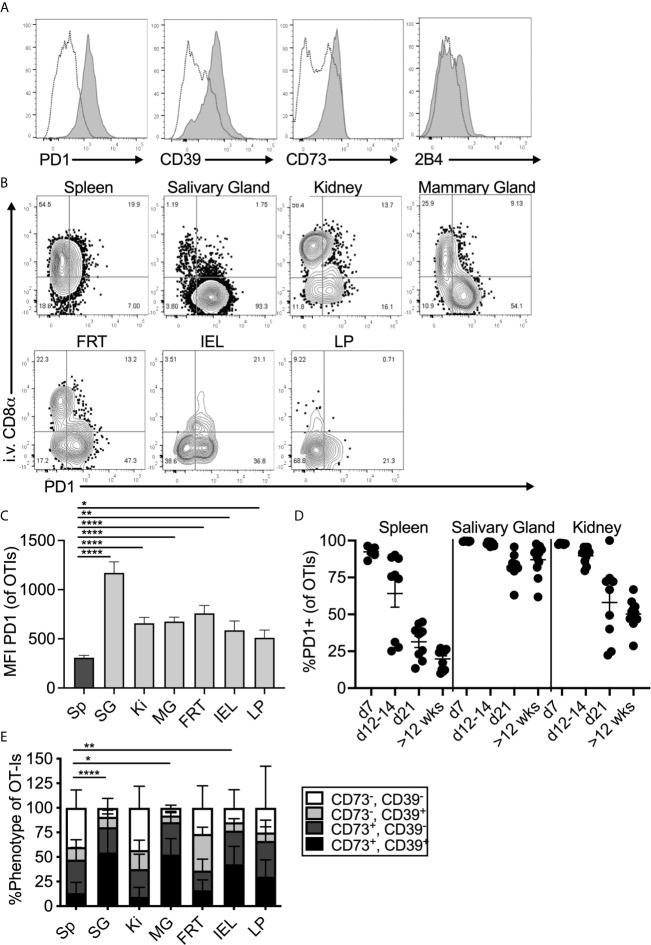
Expression of inhibitory molecules on MCMV specific T cells in the salivary gland. Mice were seeded with OT-Is and infected with MCMV-OVA. At least 3 months post infection mice were injected i.v. with fluorochrome labeled anti-CD8α antibody and sacrificed 3 minutes later. **(A)** Representative FACS plots show inhibitory molecule expression on i.v. antibody-negative salivary gland OT-Is (grey histograms) compared to total (i.v. negative and i.v. positive combined) spleen derived OT-Is from the same animal (open histograms, n=5 from 3 independent experiments). **(B)** Representative FACS plots of OT-Is isolated from the indicated organs showing PD-1 expression relative to staining by the intravenously injected CD8α antibody. **(C)** Mean fluorescence intensity of PD-1 staining on OT-Is from the indicated organs, (n=6-9 mice from 3 experiments). Data show the mean of all mice and error bars show the standard error of the mean. **(D)** Frequency of PD-1 expression on OT-Is from the spleen, salivary gland and kidney over time (n=6-11 from 2-4 independent experiments at each time point). Horizontal lines represent the mean and error bars show the standard error of the mean. **(E)** Frequency of CD73 and CD39 expression on OT-Is isolated from the indicated organs, (n=4-13 from 4 independent experiments). Data show the mean of all mice and error bars show the standard error of the mean. Statistical significance in all cases, was tested by direct comparisons between pairs of organs using paired, two-tailed student’s t-tests. For simplicity and clarity, shown are significant differences between spleen and other organs. *p ≤0.05, **p≤0.01, ***p≤0.001, ****p≤0.0001.

To study tissue-localized T cells, mice were seeded with congenically marked OT-Is one day before infection with MCMV-OVA and sacrificed more than three months post infection. In all experiments, fluorochrome labelled CD8α antibody was injected intravenously immediately prior to sacrifice to distinguish between cells in the vasculature and cells in the parenchyma of the organs. Salivary gland-localized OT-Is expressed elevated levels of PD-1 when compared to OT-Is in the spleen (**Figure 1A**, see representative gating strategy in **Supplemental Figure 1**). Surprisingly, this expression of PD-1 was replicated throughout the body on tissue-localized MCMV-specific T cells. Indeed, MCMV specific T cells in the parenchyma, but not the vasculature, of the kidney, mammary gland, female reproductive tract and the small intestine (lamina propria and intra-epithelial lymphocytes) all expressed elevated levels of PD-1, similarly to the salivary gland (**Figure 1B**). While PD-1, was expressed on nearly all cells in the salivary gland, expression was strongest on cells that expressed the T_RM_ phenotype of CD103 and CD69 (**Supplemental Figures 2A, B**). Mean fluorescence of PD-1 and the frequency of PD-1 expression was highest on T cells in the salivary gland compared to other organs (**Figure 1C** and **Supplemental Figure 2C**). Nevertheless, these levels were intermediate when directly compared to a population of functionally exhausted tumor-specific T cells (**Supplemental Figure 2D**). Notably, expression of full-length PD-1 by sorted salivary gland OT-Is was confirmed by PCR of cDNA (not shown) as previously described (46). Analyses of T cells over time showed that PD-1 expression was lost from splenic T cells within 3 weeks of infection, as expected, but sustained on salivary gland-localized T cells (**Figure 1D**, p<0.0001 at >12 weeks post infection). Kidney-localized T cells lost PD-1 expression compared to salivary gland-localized T cells (p<0.0001), but sustained higher levels of PD-1 when compared to splenic T cells (**Figure 1D**, p<0.0001).

MCMV specific CD8^+^ T cells from the salivary gland and other tissues also expressed the adenosine producing enzymes CD39 and CD73 (**Figures 1A, E**). Like PD-1, expression of CD73 and CD39 was strongest on cells that expressed CD103 and CD69 (**Supplemental Figure 2A**). Finally, OT-Is in the salivary gland that expressed CD103 also co-expressed 2B4 (CD244), as has been previously reported for T cells in other tissues (47–49)(**Figure 1A** and **Supplemental Figure 3**). In contrast, we did not detect elevated levels of CTLA4, TIM3, LAG3, or CD160 on salivary gland-localized MCMV-specific T cells when compared to their counterparts in the spleen (not shown). Importantly, a similar phenotype was evident on MCMV-specific T cells identified by tetramer staining after infection with the K181 strain of MCMV (**Supplemental Figure 4**). Therefore, this is not an OT-I or MCMV-OVA-related phenomenon.

### Inhibitory Molecule Expression on Tissue-Localized T Cells Is Antigen Independent

**Figure 2:**
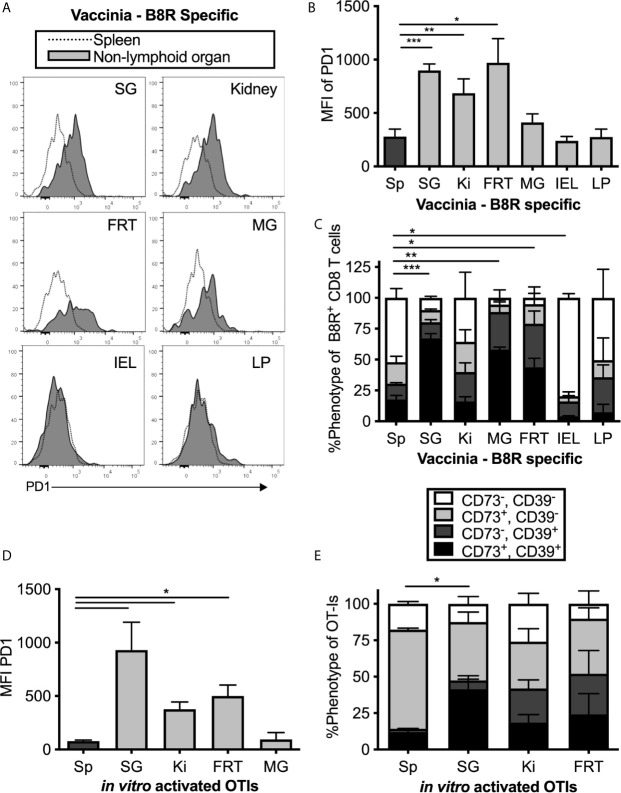
MCMV-independent and antigen-independent expression of inhibitory molecules on tissue-localized T cells. Mice were infected with Vaccinia and virus specific CD8s were identified by B8R tetramer more than 1 month post infection. **(A)** Representative FACS plots show PD-1 expression on i.v. antibody-negative B8R specific T cells in the indicated non-lymphoid organ (grey histograms) compared to all T cells in the spleen (open histograms). **(B)** Mean fluorescence intensity of PD-1 on B8R specific cells from the indicated organs. Data show the mean of n=8 mice from 2 independent experiments and error bars show the standard error of the mean. **(C)** Frequency of CD73 and CD39 expression on B8R specific T cells from the indicated organs. Data show the mean of n=3 mice from one experiment and the error bars show the standard deviation of the group. **(D)** OT-Is were activated with peptide *in vitro* for 3 days before adoptive transfer into naïve mice, which remained uninfected after transfer. OT-Is recovered from the indicated organs 2 weeks after transfer. Data show the mean fluorescence intensity of PD-1 from recovered OT-I cells (n=3 mice per group from one experiment, representative of 3 independent experiments with 11 mice total) and the error bars show the standard deviation of the group. **(E)** OT-Is from the experiment in “D” were analyzed for expression of CD73 and CD39. Shown is the frequency of CD73 and CD39 expression on OT-Is isolated from the indicated organs from n=3 mice per group. Error bars represent the standard deviation of the group. Statistical significance in **(B–E)** was tested by direct comparisons between pairs of organs using paired, two-tailed student’s t-tests. As in **Figure 1**, shown are significant differences between spleen and other organs for the MFI of PD-1 **(B, D)** or the frequency of cells expressing both CD73 and CD39 **(C, E)**. *p≤0.05, **p≤0.01, ***p≤0.001, ****p≤0.0001.

PD-1 has been identified on T_RM_ populations or tissue-localized T cells in some tissues (44, 50–53), and it is unclear whether this is associated with local antigen. However, it is well-known that PD-1 and CD39 are both expressed on recently-activated T cells and both are markers of chronic antigen exposure and functional exhaustion (54–57). The MCMV-OVA strain we used is highly attenuated for salivary gland infection compared to wild-type MCMV (40, 58) and therefore would seem unlikely to drive T cell exhaustion. Nevertheless, it does reach the salivary gland and persist there. Thus, it was possible that MCMV-specific T cells in tissues might be driven to express inhibitory molecules as a result of MCMV persistence. To test this, mice were infected with Vaccinia virus (VV), a non-persistent DNA virus, and were sacrificed at least 1 month post infection. T cells specific for the VV epitope B8R were identified by tetramer staining. Remarkably, expression of PD-1, CD39, CD73 and 2B4 was generally similar between MCMV-specific and VV-specific T cells including intermediate expression of PD-1 and co-expression of CD39 and CD73 in multiple organs (**Figures 2A–C**). These data suggest that elevated expression of these molecules is not associated with viral persistence or latency and not dependent on the type of virus or the specificity of the T cells.

To confirm the conclusion that expression of inhibitory molecules was independent of antigen persistence, uninfected mice were seeded with *in vitro-*activated OT-I T cells, which we have previously shown can become T_RM_ in the salivary gland after adoptive transfer (15). Even without an infection, the transferred OT-I T cells that reached the salivary gland, kidney and other non-lymphoid organs expressed higher levels of PD-1, CD39 and CD73 than their counterparts in the spleen (**Figures 2D, E**). Together, these data show that expression of PD-1, CD39 and CD73 is not sustained by persistent antigen, but instead is associated with tissue-localization.

### TGF-β Is Responsible for CD73 Expression, But Not CD39 or PD-1 Expression

**Figure 3:**
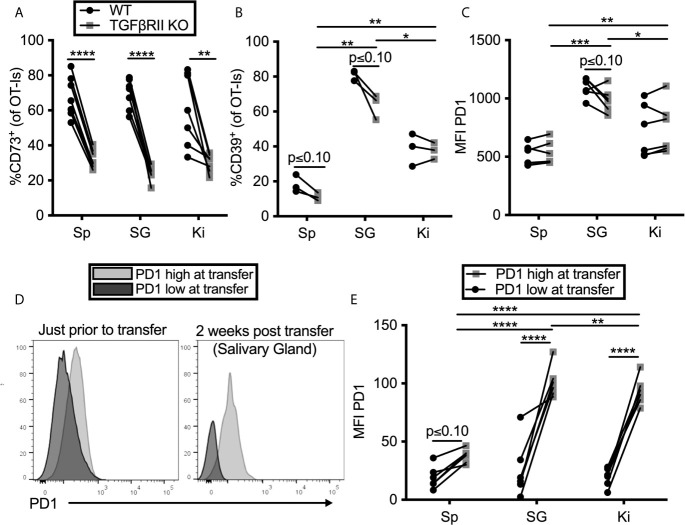
TGF-β induces CD73 expression on CD8^+^ T cells, but PD-1 is sustained by tissue localization. **(A–C)** Mice were seeded with a mixture of wild type (WT) and TGFβII receptor KO OT-Is and infected with MCMV-OVA. Mice were sacrificed at least 1 month post infection. Expression of **(A)** CD73 **(B)** CD39 and **(C)** PD-1 on OT-Is from the indicated organs. Lines connect TGFβII receptor KO OT-Is and WT OT-Is from the same mice. Data were analyzed with paired t-tests to assess differences between WT and KO cells recovered from the indicated organ of each animal and to compare cells between organs of the same animal (n=7 mice pooled from 2 independent experiments). **(D, E)** PD-1^high^ OT-Is were generated by seeding mice with RAG^-/-^ OT-Is, infecting recipients with MCMV-OVA and harvesting T cells on day 7. PD-1^low^ populations differing at the CD45 locus were generated by activating RAG^-/-^ OT-I T cells *in vitro* with peptide for three days and then resting for three days without peptide stimulation. PD-1^high^ and PD-1^low^ populations were mixed and adoptively transferred into naïve mice. **(D)** Representative FACS plots of PD-1 expression on the in *vivo* (PD-1^high^) and *in vitro* (PD-1^low^) generated T cells just before adoptive transfer and again, on i.v. antibody-negative OT-Is after recovery from the salivary gland two weeks after adoptive transfer. **(E)** Mean fluorescence intensity of PD-1 expression on OT-Is recovered from recipient mice two weeks after transfer. Lines connect the two different OT-I populations recovered from the same recipients. Data were analyzed with paired t-tests to assess differences between WT and KO cells recovered from the indicated organ of each animal and to compare cells between organs of the same animal (n=6 mice pooled from 2 independent experiments) *p ≤0.05, **p≤0.01, ***p≤0.001, ****p≤0.0001.

TGF-β is known to be important for the tissue-resident T cell program, including expression of CD103 (59–62). Moreover, CD73-expression has been previously linked to TGF-β signaling (63). Previous work from our lab has shown that TGF-β is constitutively expressed in the salivary gland, even without infection (15) and the Oxenius lab reported that it is essential for normal T_RM_ numbers in the salivary gland after MCMV infection (14). Thus, we tested the impact of TGF-β signaling on PD-1, CD39 and CD73 expression on T cells in the salivary gland and kidney. To this end, OT-I T cells lacking the TGF-β-RII were mixed with congenic wild type OT-I T cells from litter mates and co-transferred into mice that were subsequently infected with MCMV-OVA. In agreement with the data from the Oxenius lab, lack of the TGF-β-RII on OT-Is reduced their frequency in the salivary gland relative to wild-type cells and eliminated expression of CD103 (**Supplemental Figures 5A, B**). Interestingly, TGF-β-RII^-/-^ OT-Is were not under-represented in the kidney (**Supplemental Figure 5A**), where the development of the T_RM_ phenotype is much less apparent (13). Strikingly however, the frequency of CD73 on OT-Is was severely reduced on TGF-β-RII KO OT-Is compared to wild type OT-Is in all organs tested (**Figure 3A**). In contrast, the loss of TGF-β signaling only had a mild and not statistically significant impact on the expression of CD39 (**Figure 3B**) and PD-1 (**Figure 3C**).

Previous work from our lab has shown that T cells only traffic to, and become resident in, the salivary gland when they have been recently stimulated by antigen (13). This raises the question of whether the tissue environment is causing an upregulation of PD-1 or if instead, PD-1 is already expressed by recently-stimulated cells arriving in the gland and then being retained. To test this, we compared OT-I T cells that expressed high or low levels of PD-1 prior to transfer into naïve, uninfected mice. We have previously shown that activated T cells can reach the salivary gland in naïve, uninfected recipients (15). OT-I T cells lacking RAG1 were specifically used in this experiment to rule out the possibility that endogenous TCR-α chains might lead to cross-reactivity with antigens in the mouse. To generate PD-1^high^ OT-Is, we seeded mice with RAG^-/-^ OT-Is, infected the recipients with MCMV-OVA, and isolated OT-I T cells from the spleens of mice 7 days later, a time at which they express high levels of PD-1. As a comparison, we generated a PD-1^low^ population by activating congenic RAG^-/-^ OT-I T cells *in vitro* with peptide for three days and then resting the cells for three days without peptide stimulation. These two populations were mixed and co-transferred into naïve mice. The difference in PD-1 expression when these cells were mixed for transfer is shown in **Figure 3D**. Two weeks later, each population retained different levels of PD-1 in the salivary gland, kidney and spleen (**Figures 3D, E**). Most strikingly, the retention of PD-1 was significantly greater in the salivary gland and kidney in comparison with the spleen. Together, these data suggest that the tissue environments of the salivary gland and kidney promote retention of PD-1 on T cells, independently of virus or antigen, while CD73 expression is induced by TGF-β after cells arrive.

### Blocking PD-L1 Receptors Improves the Function of Salivary Gland T Cells *Ex Vivo*

**Figure 4:**
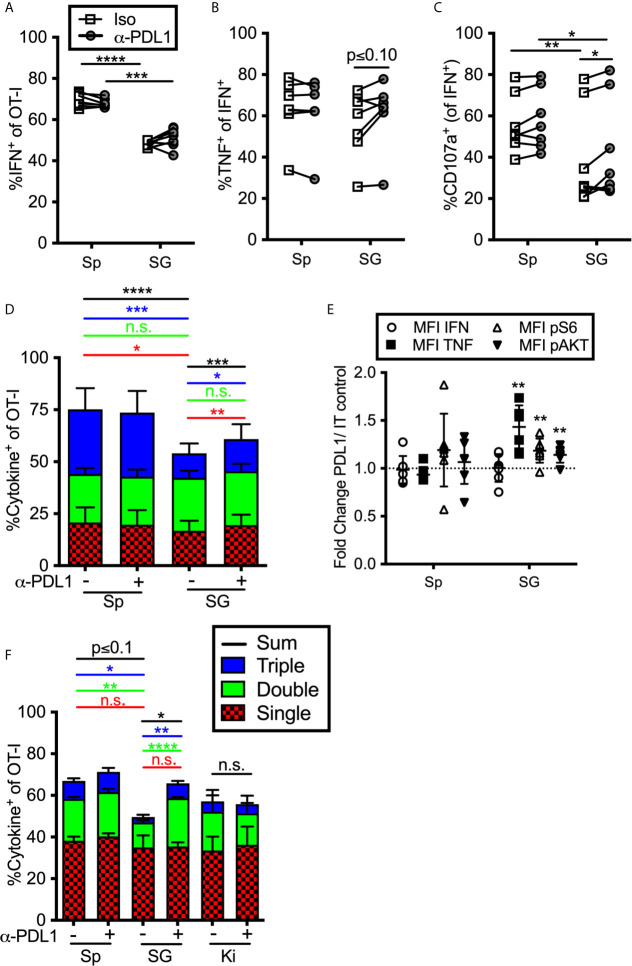
Impact of blocking PD-L1 on T cell function *ex vivo*. **(A–E)** Mice were seeded with OT-Is and infected with MCMV-OVA. At least 3 months post infection OT-Is were harvested from the indicated organs as described in the materials and methods, and stimulated for 5 hours with SIINFEKL pulsed DC2.4s, in the presence of anti-PD-L1 or isotype control antibody (iso). **(A)** Frequency of OT-Is that produced IFN-γ, or **(B)** co-produced IFN-γ and TNF-α or **(C)** produced IFN-γ and exposed CD107a during stimulation. Each line connects OT-Is from the same mouse (n=7 mice pooled from 3 independent experiments). Data were analyzed in pairwise fashion using paired, two-tailed t-tests to assess differences between cells recovered from different organs of the same mouse, or cells recovered from a single organ and restimulated with or without blocking antibody. **(D)** Boolean gating of OT-Is from **(A–C)** expressing IFN-γ, TNF-α and/or CD107a after stimulation, (n=7 from 3 independent experiments). Populations expressing 1, 2, and/or 3 functions were analyzed by paired two-tailed t-tests. **(E)** Fold change in mean fluorescence intensity of the indicated marker of anti-PD-L1 treated OT-Is relative to isotype control treated samples from samples in **(A–D)**. In order to compare the MFI of the indicated molecule across experiments, the data was plotted as the ratio between cells treated with the PD-L1 blocking antibody *vs* cells treated with the isotype control antibody. Therefore, a value of 1 would indicate no change between treated and control wells. Statistical tests were conducted using one sample t-tests between each condition and a hypothetical value of 1 (n=7 mice from 3 independent experiments). **(F)** Mice were seeded with OT-Is and infected with MCMV-OVA. At least 3 months after infection, mice were treated with anti-PD-L1 blocking antibody or isotype control antibody for 2 weeks. Lymphocytes were isolated from the indicated organs and stimulated for 5 hours with SIINFEKL pulsed DC2.4s in the presence of the same antibody that was injected into the mice. The data show Boolean gating of IFN-γ, TNF-α and CD107a expression by OT-Is after stimulation. Since cells were derived from different mice treated with isotype control antibody or PD-L1 blocking antibody, populations expressing 1, 2, and/or 3 functions were analyzed by unpaired two-tailed t-tests. Data are from n=3 mice, representative of 2 independent experiments, n=6. *p ≤0.05, **p≤0.01, ***p≤0.001, ****p≤0.0001, n.s., not significant.

To determine if constitutive inhibitory molecule expression had an impact on T cell function, we isolated OT-Is from the organs of MCMV-infected mice (>12 weeks after infection) and stimulated them *ex vivo* on peptide pulsed DC2.4s (PD-L1^+^, not shown) in the presence or absence of a PD-L1 blocking antibody. Notably, we purified these T cells through a discontinuous Percoll gradient as previously described (23). This purification and the use of antigen presenting cells (APCs) resulted in increased function compared to T cells stimulated in the salivary gland homogenate (not shown), which was the protocol used in a previous study by Hofmann and Pircher (38). Nevertheless, OT-Is isolated from the salivary gland produced IFN-γ and degranulated at significantly lower frequencies than OT-Is isolated from the spleen (**Figures 4A–C**). Preventing OT-Is from engaging with PD-L1 during antigen encounter slightly improved the production of TNF-α and degranulation (**Figures 4B, C**). Boolean gating of T cells producing IFN-γ, TNF-α and exposing CD107a revealed that including the PD-L1 blocking antibody in the stimulation culture modestly improved the overall function of the T cells from the salivary gland (**Figure 4D**). Consistent with this, T cells also exhibited an increased mean fluorescence intensity of TNF-α when PD-L1 was blocked during stimulation, relative to the same cells stimulated without PD-L1 blockade (**Figure 4E**). Moreover, blocking PD-L1 also increased the phosphorylation of molecules AKT and S6, which are downstream of TCR signaling and have been shown to be modulated by PD-1 signaling during T cell activation (64, 65). These data show that the intermediate levels of PD-1 that are constitutively present on salivary gland T cells are sufficient to reduce the strength of TCR signaling and modestly inhibit effector functions.

We considered the possibility that T cell function would be more markedly altered by treating the mice with PD-L1 blocking antibody before T cells were extracted and stimulated. To this end, we treated MCMV-infected mice (>12 weeks post-infection) with a PD-L1 blocking antibody or an isotype control antibody for two weeks. The i.p. injected blocking antibody infiltrated non-lymphoid organs and blocked PD-L1, as assessed by staining harvested tissues *ex vivo* with a fluorescently labelled version of the same anti-PD-L1 antibody clone used for blocking (**Supplemental Figure 6**). OT-Is were isolated from the spleen, salivary gland and kidney and were maintained in the presence or absence of the blocking antibody during restimulation. In this setting, blocking PD-L1 also resulted in a significant increase in cells that were double or triple positive (**Figure 4F**). These data suggest that ongoing PD-L1 signaling in the animal contributes to the reduction of T cell functionality in the salivary gland. In contrast, the function of kidney derived OT-Is was not altered by the PD-L1 blockade (**Figure 4F**), despite their expression of PD-1 (**Figures 1**–**3**).

### CD73 Blockade Improves T Cell Function in the Kidney and Spleen, But Not the Salivary Gland

**Figure 5:**
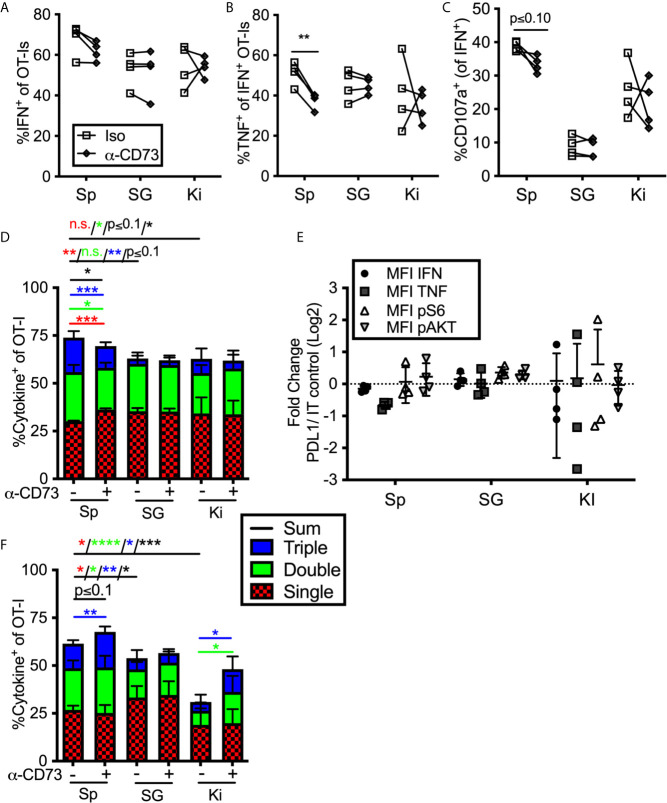
Impact of blocking CD73 on T cell function *ex vivo*. **(A–E)** Mice were seeded with OT-Is and infected with MCMV-OVA. At least 3 months post infection OT-Is were harvested from the indicated organs and stimulated for 5 hours with SIINFEKL pulsed DC2.4s in the presence of anti-CD73 or and isotype control antibody (iso). **(A)** Frequency of OT-Is that produced IFN-γ, or **(B)** co-produced IFN-γ and TNF-α or **(C)** produced IFN-γ and exposed CD107a during stimulation. Each line connects OT-Is from the same mouse. Data were analyzed in pairwise fashion using paired, two-tailed t-tests to assess differences between cells recovered from different organs of the same mouse, or cells recovered from a single organ and restimulated with or without blocking antibody, (n=4 from 2 independent experiments). **(D)** Boolean gating of OT-Is expressing IFN-γ, TNF-α and/or CD107a after stimulation. Populations expressing 1, 2, and/or 3 functions were analyzed by paired two-tailed t-tests. (n=4 from 2 independent experiments). **(E)** Fold change in mean fluorescence intensity of the indicated marker of anti-CD73 treated OT-Is relative to isotype control antibody treated controls from the cells shown in **(A–C)**. As in **Figure 4**, the MFI of the indicated molecules were compared across experiments as the ratio between cells treated with the blocking antibody *vs* cells treated with the isotype control antibody. Therefore, a value of 1 indicates no change between treated and control wells. Statistical comparisons were conducted using one sample t-tests between each condition and a hypothetical value of 1 (n=4 from 2 independent experiments). **(F)** Mice were seeded with OT-Is and infected with MCMV-OVA. At least 3 months after infection, mice were treated with anti-CD73 blocking antibody or isotype control for 2 weeks. Lymphocytes were isolated from the indicated organs and stimulated for 5 hours with SIINFEKL pulsed DC2.4s in the presence of the same antibody that was injected into the mice. Shown is Boolean gating of OT-Is expressing IFN-γ, TNF-α and/or CD107a after stimulation. Since cells were derived from different mice treated with isotype control antibody or CD73 blocking antibody, populations expressing 1, 2, and/or 3 functions were analyzed by unpaired two-tailed t-tests. Data are from n=6 mice from 2 independent experiments). *p ≤0.05, **p≤0.01, ***p≤0.001, ****p≤0.0001, n.s., not significant.

To assess the impact of enzyme-mediated adenosine production, we repeated the previous set of experiments using a CD73 blocking antibody. Adding anti-CD73 antibody during *ex vivo* stimulation failed to improve the frequency of cells from any tissue to produce cytokines or degranulate, and in fact, slightly reduced the function of OT-Is from the spleen (**Figures 5A–C**) resulting in a significant increase in cells with only a single function after stimulation and a decrease of cells with two or three functional responses (**Figure 5D**). Similarly, blocking CD73 failed to increase the mean fluorescence of cytokines produced by stimulated cells or the phosphorylation of AKT and S6 (**Figure 5E**). Thus, CD73 blockade did not seem to impact the strength of TCR signaling or increase effector functions upon *ex vivo* stimulation. Interestingly however, when anti-CD73 was administered to mice for 2 weeks prior to T cell isolation and *in vitro* stimulation, OT-I T cells from the kidney and spleen, but not the salivary gland, displayed improved function (**Figure 5F**). Adenosine production *via* CD39 and CD73 is dependent on the presence of extracellular ATP, which is released upon cell death and stress. Therefore, we wondered if a CD73 blockade would have more of an impact on salivary gland-localized T cells at early times post infection, when there is still virus replicating in the salivary gland. However, blockade of CD73 from day 7 to 21 after infection also failed to improve the function of salivary gland T cells (not shown). Together, these data suggest that T cells localized to the salivary gland, spleen and kidney were differentially impacted by PD-1 and adenosine production: salivary gland localized T cells were inhibited by PD-1 (**Figure 4**), while T cells in the kidney were inhibited by CD73 (**Figure 5F**).

### Inhibitory Molecules Don’t Influence Cell Number or Phenotype or Viral Control in the Salivary Gland

**Figure 6:**
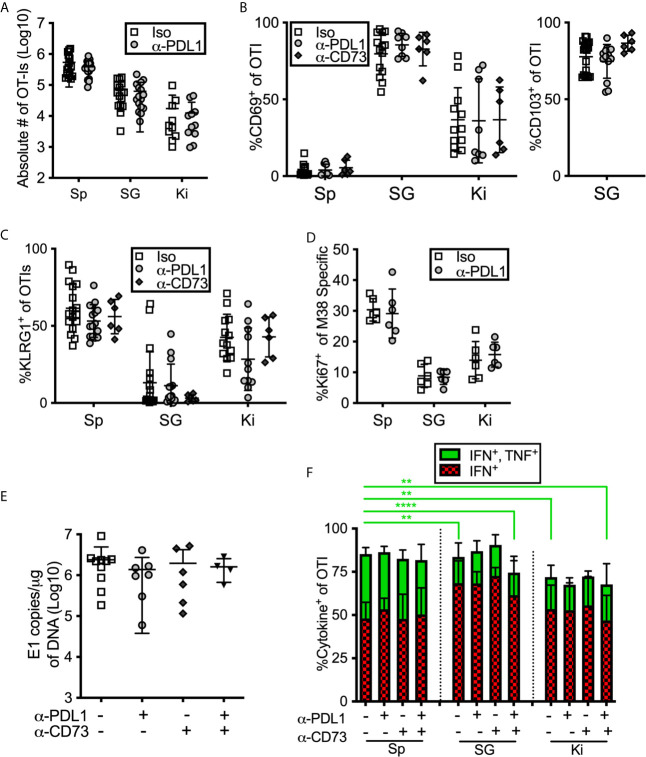
Blocking PD-L1 or CD73 has minimal impact on in *vivo* maintenance, phenotype or function of T cells. **(A–C)** Mice were seeded with OT-Is and infected with MCMV-OVA for at least 3 months, and then treated with the indicated blocking antibodies or isotype control (iso) for 2 weeks before sacrifice. The data show cells recovered from the indicated organs after antibody treatment including: **(A)** the absolute numbers of OT-Is, **(B)** the frequency of CD69 and CD103 expression by OT-Is, and **(C)** the frequency of KLRG-1 expression by OT-Is, (n=18 from 5 independent experiments). **(D)** Mice were infected with wild type MCMV and treated with anti-PD-L1 blocking antibody for one week starting on day 7 of infection. Mice were sacrificed on day 14 and MCMV specific CD8s were identified by tetramer staining. The data show the frequency of Ki-67 expression among M38-specific CD8s, (n=6 from 2 independent experiments). **(E)** Mice were infected with wild type MCMV and treated with the indicated blocking antibodies for two weeks starting on day 7 of infection. Mice were sacrificed on day 21 and viral DNA load was determined in the indicated organs by qPCR. Shown are the copies of viral genome per ug of DNA, (n=6 from 2 independent experiments). **(F)** Mice were seeded with OT-Is and infected with MCMV-OVA for at least 3 months, and then treated with the indicated blocking antibodies for 2 weeks. Following antibody treatment, mice were injected with SIINFEKL peptide (200 μg i.v and 50 μg directly into the salivary gland). Six hours after peptide injection, mice were sacrificed and organs were collected and processed in media containing brefeldin A. Shown is the frequency of IFN-γ and TNF-α producing OT-Is that were recovered. For comparison of cells from different organs of the same mice, paired two-tailed t-tests were used. For comparison of cells in the same organ but from different mice with different treatments (e.g. blockade or isotype control antibody) populations were analyzed by unpaired two-tailed t-tests. Data are from n=6 mice from 2 independent experiments. *p ≤0.05, **p≤0.01, ***p≤0.001, ****p≤0.0001.

During the course of these experiments, we were surprised to note that PD-L1 or CD73 blockade had no impact on the number of antigen specific CD8^+^ T cells or on the phenotype of those cells in mice that had been infected for more than 12 weeks with MCMV (**Figures 6A–C**). Similarly, there was no significant impact on OT-I number or phenotype when mice were treated with blocking antibodies targeting PD-L1 or CD73 – separately or in combination – between day 7 and day 21 of infection (**Supplemental Figures 7A–C**). We have previously shown that MCMV specific CD8 T cells in the salivary gland stop dividing earlier than their counterparts in the spleen (13). However, blocking PD-L1 did not reverse this result (**Figure 6D**). Finally, the salivary gland is considered the primary site of MCMV persistence in mice and CD8^+^ T cells selectively fail to control the virus in this organ (8), at least in part due to viral reduction of MHC-I antigen presentation on infected cells (11, 37) and the production of IL-10 (66). We wondered whether the inhibitory molecules expressed by salivary gland-localized T cells also contributed to their inability to clear MCMV from this organ. Therefore, we assessed the impact of PD-L1 or CD73 blockade on viral load in the salivary gland. However, neither viral genome copies (**Figure 6E**) nor viral replication (**Supplemental Figure 7E**) in the salivary gland were impacted by antibody blockades at early times after infection. Depleting CD4^+^ T cells, which are known to play a role in controlling viral replication in the salivary gland, also did not reveal an impact of the PD-L1 blockade (**Supplemental Figures 7D, E**).

### Inhibitory Molecules Don’t Improve Salivary Gland T Cell Function After Intravenous Peptide Injection

Previous work showed that salivary gland-localized T cells were more functional when stimulated *in vivo* by intravenously injected peptide, compared to assays in which those T cells were isolated and stimulated *ex vivo* (38). In agreement with this work, the majority of salivary-localized CD8^+^ T cells (~80%) produced IFN-γ, with or without TNF-α, upon i.v. peptide injection (**Figure 6F**). However, this approach did not fully rescue T cell function. Cells in the salivary gland and kidney were still less likely to produce both IFN-γ and TNF-α when compared with their counterparts from the spleen, and blockade of either PD-L1, CD73 or both, for two weeks prior to peptide injection did not change these results (**Figure 6F**). It was possible that i.v. peptide injection drove T cells to migrate into the salivary gland, which could obscure the function of T cells that had been lodged in the salivary gland. To test this, we simultaneously injected both CFSE and peptide intravenously. We have previously shown that intravenous injection of CFSE labels CD8^+^ T cells in circulation, but not the salivary gland (13). Importantly, peptide injection did not lead to an influx of CFSE-labeled CD8^+^ T cells into the salivary gland or kidney during the course of the experiment (**Supplemental Figure 7F**), showing that any cytokine expressing cells identified in this assay were lodged in the tissue at the time of peptide injection.

### Intraglandular Injections of PD-L1-Expressing Targets Reveals a Functional Role of PD-1 on Salivary Gland T Cells *In Vivo*

**Figure 7:**
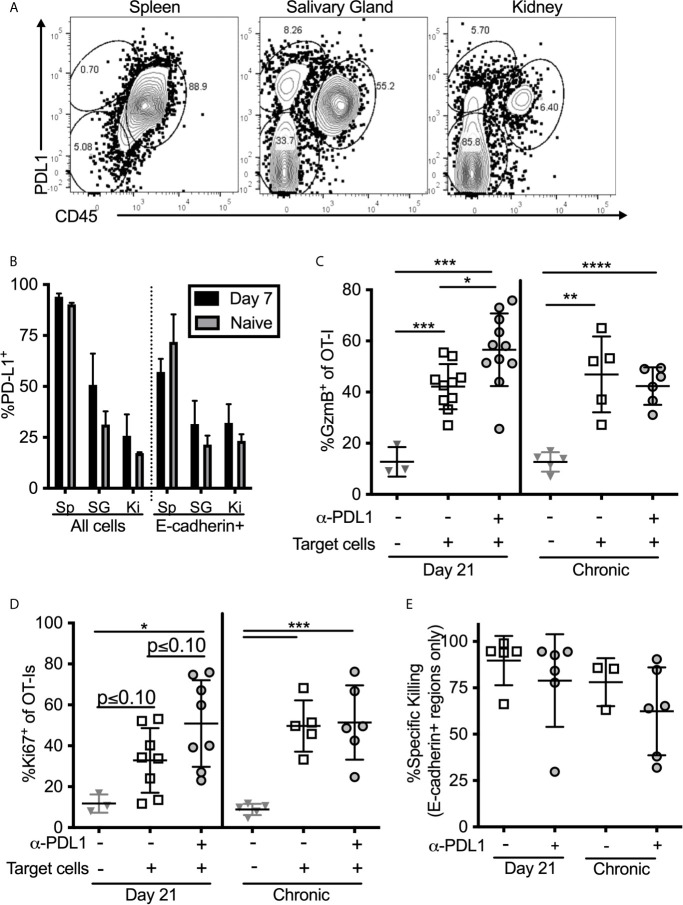
T cell stimulation by PD-L1 expressing target cells. **(A, B)** Organs from naïve mice and mice infected with wildtype MCMV for 7 days were homogenized as described in the methods, and directly stained for PD-L1 expression without Percoll isolation. **(A)** Representative FACS plots of PD-L1 expression on all cells recovered from salivary glands. **(B)** Frequency of PD-L1 expression on different subtypes of cells in the indicated organs. Data are representative of 3 mice per group. **(C–E)** Mice bearing OT-Is and infected with MCMV-OVA for either 21 days, or more than 12 weeks, received the PD-L1 blocking antibody or an isotype control antibody for two weeks. At the end of the blocking period, splenocytes from naïve mice were pulsed with either SIINFEKL or an irrelevant peptide (B8R) and labelled with CellTrace Far Red or CFSE respectively. Target cells were injected i.v. and intra-glandularly into 48 hours after target cell injection, tissues were harvested for analyses. Half of the harvested organs were used to extract lymphocytes for FACS analysis and half were frozen for immunofluorescent histology. Shown is the frequency of **(C)** granzyme B expression and **(D)** Ki-67 expression by salivary gland localized OT-Is as assessed by FACS. In all cases, data were analyzed in pairwise fashion between groups of mice treated or not with the PD-L1 blocking antibody using unpaired, two-tailed t-tests. **(E)** Specific killing of peptide pulsed splenocytes contained withing E-cadherin^+^ regions of the salivary gland as assessed by histology (n=6-11 mice from 4 independent experiments). *p ≤0.05, **p≤0.01, ***p≤0.001, ****p≤0.0001.

One possible explanation for the discrepancy between our *ex vivo* stimulation results (**Figures 4**, **5**) and the results obtained after *in vivo* injection of peptide (**Figure 6E**) may be that responding T cells are encountering antigen on cells that lack PD-L1 *in vivo*. Indeed, PD-L1 is not expressed as highly in the salivary gland and kidney as it is in the spleen, and the cells that do express PD-L1 are primarily (but not exclusively) of hematopoietic origin (**Figures 7A, B**). Moreover, only a minor fraction of E-cadherin expressing epithelial cells in the salivary gland expressed PD-L1 in both infected and naïve mice (**Figure 7B**). Since acinar epithelial cells are an important site of MCMV replication and persistence, these data suggest that many of these targets may lack PD-L1. Therefore, we tested whether PD-1 altered the ability of salivary gland-localized T cells to kill PD-L1-expressing targets. Through multiple experiments, we were unable to obtain sufficient numbers of T cells from the salivary gland to make *ex vivo* killing assays reproducible. Therefore, we pulsed splenocytes with the SIINFEKL peptide and injected them directly the salivary gland (intraglandular injection – i.g.) to assess killing of these targets. Mice were sacrificed 48 hours after i.g. injections. This protocol allowed us to assess the activation of the salivary gland-localized OT-I T cells and specific killing of the peptide-pulsed targets, but did not allow for the assessment of cytokine production by the T cells. First, we noted that salivary gland T cells expressed significantly lower levels of granzyme B compared to their counterparts in the spleen (**Supplemental Figure 8A**). However, OT-Is responded to the injection of peptide-pulsed targets by robustly upregulating granzyme B (**Figure 7C**) and by upregulating the division marker Ki-67 (**Figure 7D**). Blocking PD-L1 significantly increased the upregulation of granzyme B (**Figure 7C**) and seemed to enhance Ki-67 expression (**Figure 7D**) by T cells in mice that had been infected for 21 days, but not in mice that were infected for more than 12 weeks. Treating mice with both anti-CD73 and anti-PD-L1 had no additional impact on granzyme B or division (**Supplemental Figures 8A, B**).

### Specific Killing of PD-L1-Expressing Targets Within the Salivary Gland Is Unaffected by PD-L1 Blockade

To assess specific killing of cells injected into the gland, we labeled SIINFEKL-pulsed splenocytes with CellTrace Far Red dye and mixed these target cells with an equal number of cells labeled with CFSE, but loaded with the B8R peptide from VV as a control. Specific killing of SIINFEKL-pulsed targets as assessed by flow cytometry was low and quite variable (**Supplemental Figure 8C**). In contrast, killing of intravenously-injected targets in the spleen was nearly 100% in all mice. However, histological analyses of injected cells revealed that their dispersal into the salivary gland was very uneven and that they preferentially accumulated in the channels between lobules of the gland, where T cells were not identified (**Supplemental Figure 8D**). In these sites, as might be expected, target cells loaded with the SIINFEKL peptide were not effectively killed (**Supplemental Figure 8D**). These data suggest that the low overall killing of injected cells as assessed by flow cytometry, may relate to the uneven distribution of targets and the presence of targets in T cell-free channels. Remarkably however, SIINFEKL-loaded target cells were obviously reduced in the E-cadherin-expressing areas of the gland (**Supplemental Figure 8D**, right panels). We have previously shown that MCMV specific T_RM_ accumulate in and around these E-cadherin-positive cells (13). Therefore, to assess the true ability of salivary gland T cells to kill the injected targets, we analyzed specific killing histologically, and only counted target or control cells that had infiltrated regions of the salivary gland that expressed E-cadherin, indicating epithelial cells. Using this approach, the data show that target cells localized to E-cadherin-positive regions were killed more effectively, but this was unchanged by PD-L1 blockade (**Figure 7E**). It is important to note however, that the levels of killing were still below those found within the spleen (compare to **Supplemental Figure 8C**). Together, our data show while blocking PD-L1 *in vivo* improves the effector functions of salivary gland-localized T cells, including improved cytokine production, degranulation and granzyme B expression, it does not impact the ability of these T cells to kill peptide-loaded splenocytes that express PD-L1. Moreover, the most relevant acinar epithelial cells rarely express PD-L1.

Collectively, our data suggest that tissue-localized T cells express inhibitory molecules as part of tissue residency. Salivary gland-localized T cells are modestly inhibited by PD-1 expression *in vivo*. In contrast, kidney-localized T cells were unaffected by PD-1, but modestly inhibited by CD73.

## Discussion

The salivary gland and kidneys are major sites of shedding for CMV and other viruses, and important tissues for viral spread to new hosts. In humans and mice, CMV replicates persistently in both tissues and infectious virus can be detected in the saliva of mice and both saliva and urine of humans for prolonged periods of time (4, 33, 67). It is possible that tissue-localized immune regulation is exploited by CMV and other viruses to persist in these tissues. Our data revealed that expression of inhibitory molecules PD-1, CD73 and CD39 is antigen-independent and linked to tissue localization. T cells in the salivary gland were particularly likely to express all 3 molecules and were inhibited by PD-1. In contrast, kidney localized T cells were less likely to express these molecules, but were inhibited by CD73.

It is well-known that CD8^+^ T cells are not effective at controlling MCMV in the salivary gland (8) and that CD4^+^ T cells are needed in the salivary gland to control replicating MCMV, presumably through their production of IFN-γ (37, 68). In fact, CD8^+^ T cells in the salivary gland appear to develop into resting, tissue-resident cells despite the ongoing viral replication in this tissue. We previously showed that CD8^+^ T cells that entered the salivary gland stopped dividing earlier than circulating CD8^+^ T cells in the same mice and differentiated into T_RM_ cells during the height of viral replication in this tissue (13). The development of T_RM_ occurred in a TGF-β-dependent manner (14) and (**Supplemental Figures 5A, B**) and the pace of T_RM_ formation was completely unaffected by virus in the salivary gland (15). Our data shown here indicate that that these T cells also exhibit reduced granzyme B expression relative to their counterparts in the spleen (**Supplemental Figure 8A**). Thus, these cells rapidly develop the phenotype that appears to be resting despite the ongoing viral replication. This finding suggests that the T cell behavior is partly the result of salivary gland localization, rather than the MCMV infection. Consistent with this, CD8^+^ T cells responding to Vaccinia virus, or that were activated *in vitro* also expressed PD-1, CD73 and CD39 in the salivary gland (**Figure 2**). This is likely relevant across infections since activated T cells migrate freely into the salivary gland where they become T_RM_ (13–15, 38, 69).

Our data further suggest that PD-1, CD73 and CD39 expression was governed by tissue localization rather than antigen. For PD-1, expression was retained on PD-1^+^ T cells that entered the salivary gland, but not induced by antigen, tissue localization or TGF-β signaling (**Figures 2** **,** **3**). This extends our previous work showing that virus-specific T cells that had migrated into a tumor retained an intermediate level of PD-1 in an antigen-independent manner (46), a finding that has been replicated in humans (70). Other labs have found that T_RM_ cells can express PD-1 in other tissues (44, 50–53), and the Lukacher lab showed that PD-1 expression on brain T_RM_ was retained in an antigen-independent manner (44). Collectively, these data are striking since PD-1 is well-described to be silenced in the absence of ongoing antigen stimulation. Repression of PD-1 is induced by the B-lymphocyte-induced maturation protein-1 (Blimp-1), which alters transcription factor binding in the *pdcd1* (PD-1) gene promoter and promotes repressive histone methylation (71, 72) unless T cells are exposed to chronic antigen (73, 74). Thus, together these data imply that repression of PD-1 fails to occur in some non-lymphoid tissues and it will be interesting to determine whether the repressive methylation of the *pdcd1* promoter is impaired in salivary gland or kidney-localized T cells, or T cells in other tissues.

It was also surprising that tissue-localized T cells, particularly those in the salivary gland, expressed both CD39 and CD73. Both regulatory T cells (T_REG_) and T_H_17 cells are known to express CD39 and CD73 (75–78) and this expression is associated with immune suppressive function of both populations through the production of adenosine in the environment. Expression of CD73 on CD8^+^ T cells in the salivary gland and kidney (as well as the spleen) was induced by TGF-β signaling (**Figure 3**), which is consistent with previous work on T_REG_ (63). Since TGF-β is also vital for the formation of T_RM_ (14, 59–62) and (**Supplemental Figures 5A, B**), these data link the T_RM_ program to the expression of the suppressive CD73 molecule. Interestingly, TGF-β was not required for CD39 expression in the salivary gland T cells despite being implicated in the expression of CD39 on T_H_17 cells (77). Thus, together, our data suggest that both PD-1 and CD73 expression by CD8^+^ cells was governed by tissue localization in distinct ways.

It was previously shown by Hofmann and Pircher that T cells recovered from the salivary gland functioned poorly in *ex vivo* stimulation assays (38). Likewise, when compared to T cells recovered from the spleen or lung with the same TCR, a smaller percentage of CD8^+^ T cells from the salivary gland made IFN-γ or degranulated upon stimulation (14) and (**Figures 4A–C**). These data raised the possibility that these inhibitory molecules might be contributing to the reduced T cell function and thus, the failure of CD8^+^ T cells to control MCMV in the salivary gland. However, blockade of PD-L1 and/or CD73 had no effect on viral load in the salivary gland (**Figure 6D**). Nevertheless, PD-L1 blockade *in vivo* prior to T cell recovery improved the function of these T cells *ex vivo* (**Figure 4F**). Interestingly, the Pircher lab also showed that salivary gland-localized T cells could better secrete IFN-γ when the stimulating peptide was injected intravenously (38). We also found that more T cells produced cytokine after i.v. injection of the peptide (**Figure 6F**). However, PD-L1 blockade had no discernable effect in this setting. It must be noted that this approach will load peptide on a multitude of potential targets and antigen presenting cells. In the salivary gland and kidney, most of the non-hematopoietic cells lack PD-L1 (**Figure 7A**) and thus may not be able to engage this inhibitory pathway. For this reason, we also tested direct intra-glandular injections of peptide-pulsed splenocytes, nearly all of which express PD-L1 (**Figure 7A**). These data revealed that PD-L1 blockade could modestly improve the induction of Granzyme B and possibly enhance T cell proliferation after stimulation (**Figures 7C, D**). However, these effects were only evident shortly after the primary infection and PD-L1-expressing targets were killed equally well within the salivary gland with or without PD-L1 blockade (**Figures 7C, E**). These data could suggest that PD-1 is not markedly impairing the function of T cells within the salivary gland environment, especially at late times post infection. However, it is also possible that PD-1 is part of a redundant set of inhibitory mechanisms that collectively impair T cell function and/or that the impact of PD-1 is relatively subtle and only apparent when the target cells are hard to kill. This last idea is intriguing since viral immune evasion of MHC-I antigen presentation makes infected cells hard to kill (79–82). While we did not observe an effect of PD-L1 blockade on viral titers in the salivary gland, our data suggest that this pathway will contribute to T cell inhibition in other settings.

We believe that one of the most important additional implications these data is the notion that *ex vivo* assays for T cell function may be markedly affected by the tissue from which the cells are recovered and may not faithfully report on the true T cell function. We have found that purifying the salivary gland T cells through a discontinuous Percoll layer (see materials and methods) improved their function compared to stimulations of T cells within the salivary gland homogenate (not shown), which was the approach used by Hoffman and Pircher (38). Moreover, the choice of APCs used in the *in vitro* assay likely plays a significant role in the outcome. In this study, as in previous work from the Lukacher lab (44), we used DC2.4 cells as APCs. These cells are PD-L1^+^ and are considered to be a dendritic cell line. However, the impact of PD-1 was less evident when we used a fibroblast cell line (M2-10B4) that was treated with IFN-γ to induce expression of PD-L1 (not shown). Despite confirmation of PD-L1 on these cells, there are likely a large number of differences in the expression of antigen presentation and co-stimulation machinery when compared to DC2.4s. For these reasons, it will be critical to test the function of non-lymphoid T cells after purification and with a variety of APCs, or *in vivo* whenever possible, before drawing conclusions about their abilities.

We also tested the function of CD73 in suppressing T cell function. Together, CD73 and CD39 convert extracellular ATP to adenosine, which is an immune suppressive mechanism used by regulatory T cells (75, 76). In contrast to blockade of the PD-1/PD-L1 pathway, we did not observe an *in vitro* or *in vivo* effect of blocking CD73 on salivary gland T cells. However, we did observe improved function of T cells derived from the kidneys and spleen. Again, restoration of T cell function required pre-treatment of the mice with CD73 blocking antibody (**Figures 6D, F**), indicating that CD73-induced suppression was occurring *in vivo* and not an artifact of the culture conditions. This result is striking since extracellular adenosine, particularly adenosine generated by CD73, is well-known to play critical physiological roles in the kidney (83). Indeed, CD73 enzymatic activity has been reported to be exceptionally high in kidneys (84, 85). Therefore, it is an intriguing possibility that the physiological function of CD73 in the kidney results in constitutive adenosine-driven suppression of T cells in this tissue and in contrast to other tissues. Much less is known about the function of CD73 and extracellular adenosine in the salivary gland. It is possible that extracellular ATP was rarely released in our experimental conditions and therefore that CD39 and CD73 rarely converted it to adenosine. However, again, there may be settings in which this pathway is engaged, resulting in adenosine-mediated inhibition of local T cells. Future work will need to address these issues.

Overall, our data show that the inhibitory molecules PD-1, CD73 and CD39 are part of tissue-localization for T cells in the salivary gland and kidney and are sustained (PD-1) or expressed (CD73) in an antigen-independent manner. The expression of PD-1, but not CD73, resulted in inhibition of T cells in the salivary gland, but the impact was modest and context dependent and did not prevent T cells from killing PD-L1 expressing targets within the salivary gland environment. In contrast, kidney localized T cells were inhibited by CD73, but not PD-1.

## Data Availability Statement

The raw data supporting the conclusions of this article will be made available by the authors, without undue reservation.

## Ethics Statement

The animal study was reviewed and approved by the Thomas Jefferson University Institutional Animal Care and Use Committee.

## Author Contributions

CJS and CMS conceived of the project and designed the experiments. CJS conducted the experiments. CJS and CMS interpreted the results and wrote the manuscript. All authors contributed to the article and approved the submitted version.

## Funding

This work was supported by the grants AI106810 an AI146235 from the NIH awarded to CMS. The funding agency had no input into the experimental design or data analyses and interpretation.

## Conflict of Interest

The authors declare that the research was conducted in the absence of any commercial or financial relationships that could be construed as a potential conflict of interest.
