# Supplementary material for: Inhibitory Molecules PD-1, CD73 and CD39 Are Expressed by CD8+ T Cells in a Tissue-Dependent Manner and Can Inhibit T Cell Responses to Stimulation
Source: Front Immunol. 2021 Jul 15;12:704862. doi: 10.3389/fimmu.2021.704862 (PMC8320728; doi:10.3389/fimmu.2021.704862)
Supplement: Supplementary file 1 [file DataSheet_1.pdf]

Supplemental Figure 1

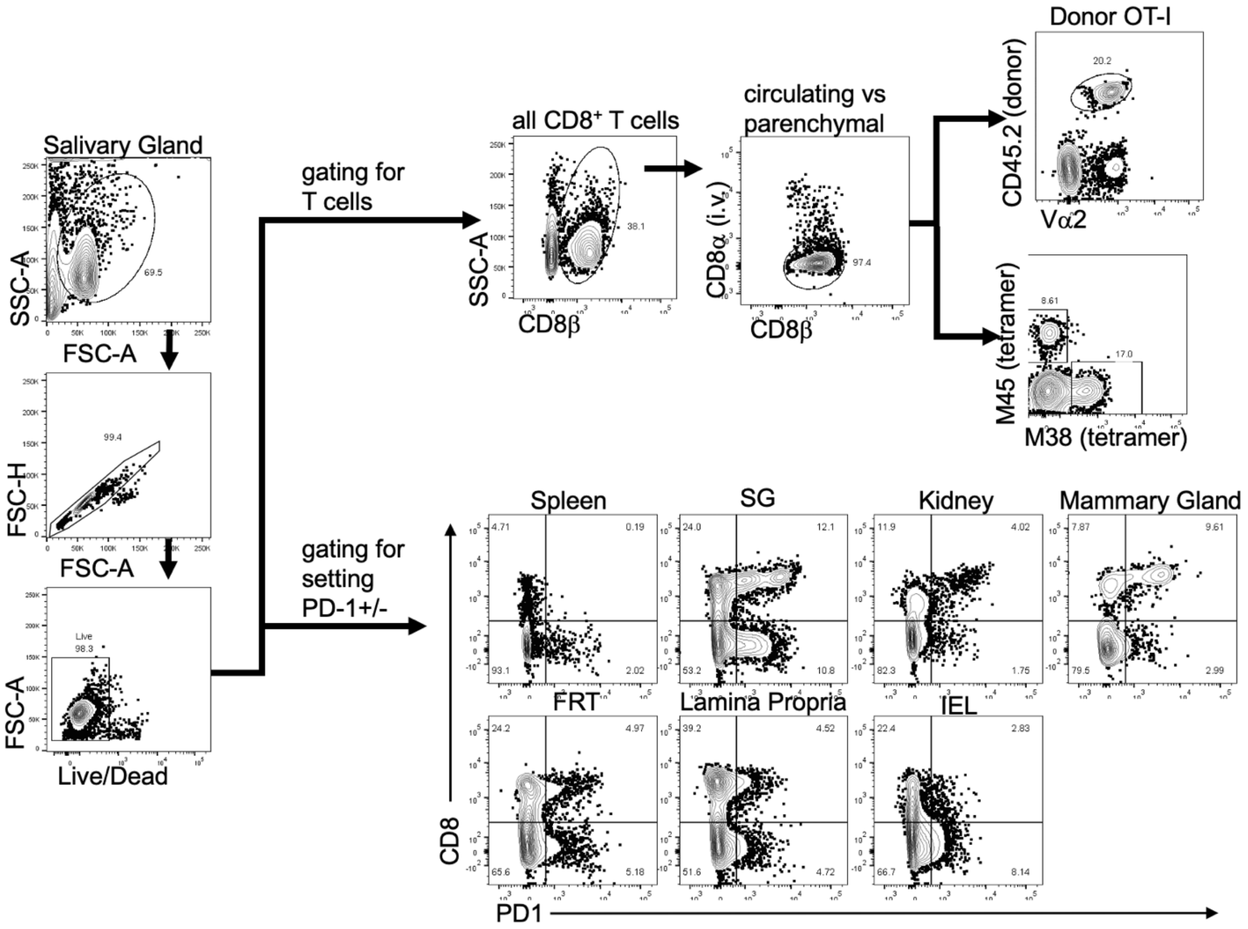

Figure S1: Representative Gating Strategy

Gating for T cells in the salivary gland is shown. Gating was identical for all organs assessed except for spleens, in which the i.v. antibody stain was not used prior to selection of OT-I or tetramer-binding T cells. For setting PD-1 gates, we used the total, live cell population (bottom panels), which contained both PD-1$^+$ and PD-1$^-$ cells in all tissues.

Supplemental Figure 2

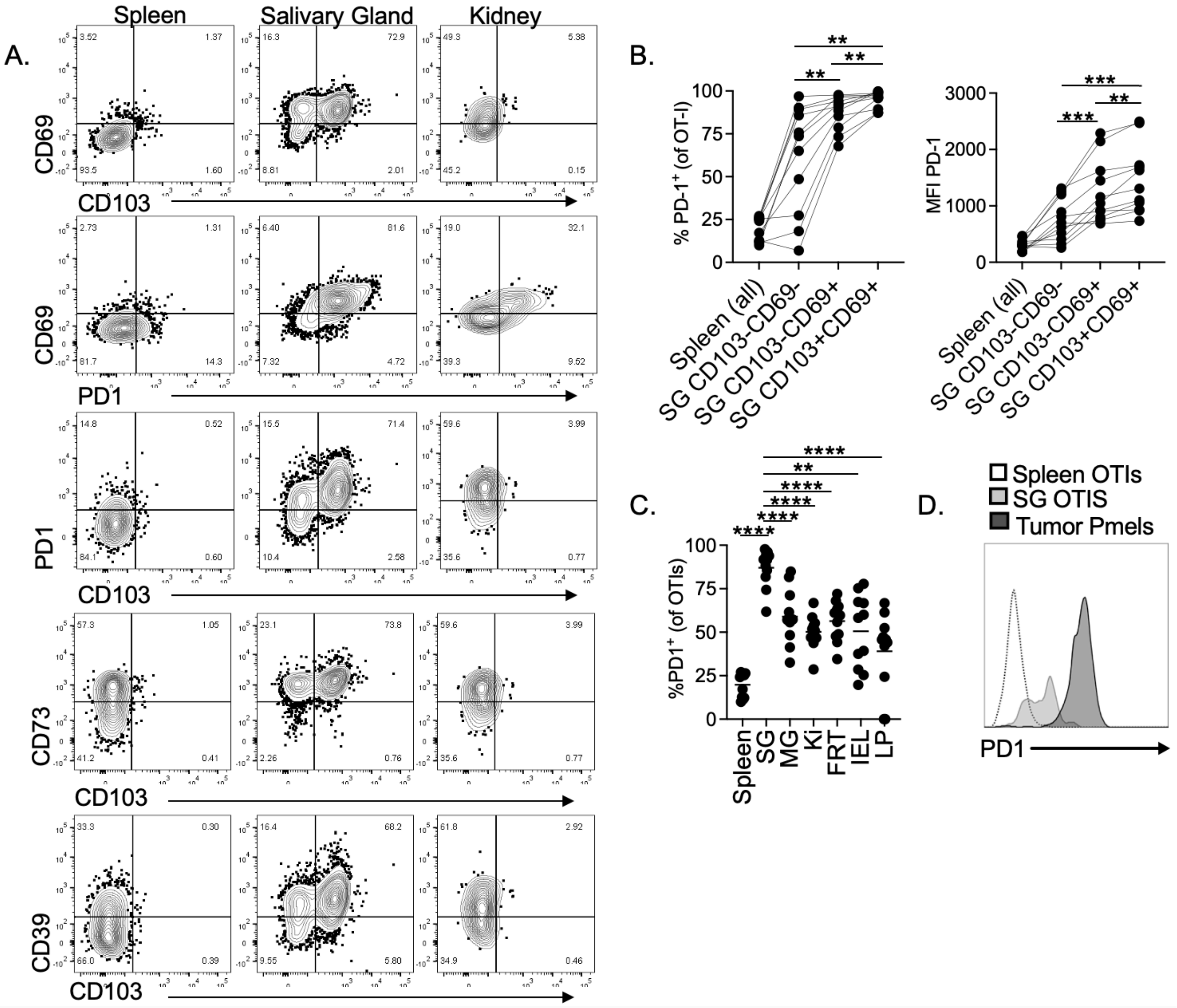

Figure S2: Phenotype of T cells in non-lymphoid organs

A) Shown is the expression of the indicated markers on OT-I T cells recovered from the indicated organs. Data are representative of n=14 mice from 4-5 independent experiments, as in Figure 1. B) Shown is the frequency (left) and MFI (right) of PD-1 expression on OT-Is recovered from the spleen and salivary gland is shown in comparison with the T_{RM} markers CD103 and CD69 (n=11 mice from 4 experiments). The data combines PD-1 staining by 2 different fluorophores, but lines connect matched samples. C) Shown is the frequency of PD-1 expression on OT-I T cells recovered from the indicated organs (n=11 mice from 4 experiments). Horizontal lines represent the median value. For B and C, statistical significance was tested by direct comparisons between each phenotype within the salivary gland (B) or between cells in each tissue of the same animals (C) using paired, two-tailed student's t-tests. * = p ≤0.05, ** = p≤0.01, *** = p≤0.001, **** = p≤0.0001. D) OT-Is and Pmel-Is were co-transferred into naïve C57BL/6 mice one day before implantation of the B16F0 melanoma cell line. Recipients were co-infected with MCMV-SL8 and MCMV-gp100 five days after tumor implantation as described previously (Erkes et al., 2017). Mice were sacrificed when the tumor reached 100mm^2. Representative FACS plot shows PD-1 expression by OT-Is isolated from the spleen and salivary gland compared to Pmel-Is isolated from the tumor. Data are representative of n=7 mice from 3 independent experiments.

Supplemental Figure 3

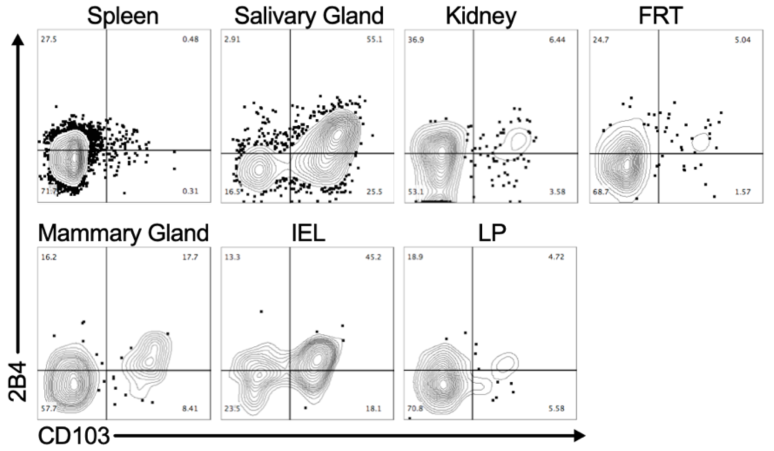

Figure S3: 2B4 and CD103 expression
Shown is the expression of the indicated markers on OT-I T cells recovered from the indicated organs.

Supplemental Figure 4

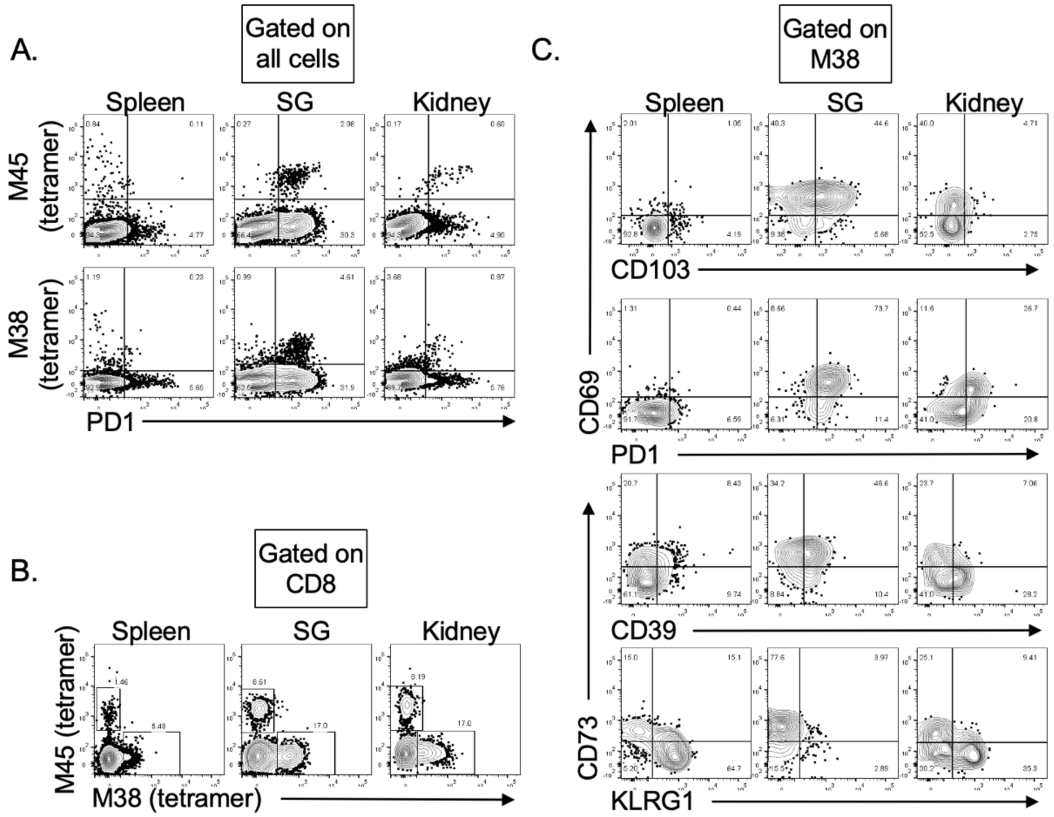

Figure S4: PD-1, CD39 and CD73 expression by polyclonal MCMV-specific T cells detected by tetramer binding. A) Shown is the tetramer staining by PD-1 expression on all cells recovered from the spleen, salivary gland and kidney, >12 weeks after infection. B) Shown is the tetramer staining on $CD8^+$ T cells. C) Shown is the phenotype of M38-specific T cells recovered from the indicated organs and co-stained with the indicated markers. As in the main manuscript, spleen data shows all $CD8^+$ T cells while salivary gland and kidney T cells were gated on i.v. antibody-negative cells.

Supplemental Figure 5

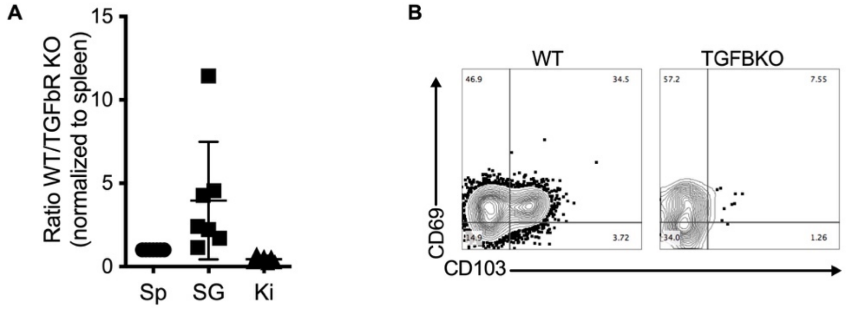

Figure S5: The TGFβII receptor is required for salivary gland localization and CD103 expression. Mice were seeded with a mixture of wild type (WT) and TGFβII receptor KO OT-Is and infected with MCMV-OVA. Mice were sacrificed at least 1 month post infection. A) Ratio of WT OT-Is to TGFβII receptor KO OT-Is in the indicated organ. Data are pooled from n=7 mice from 2 independent experiments. B) Expression of CD69 and CD103 on WT and TGFβII receptor KO OT-Is in the salivary gland.

Supplemental Figure 6

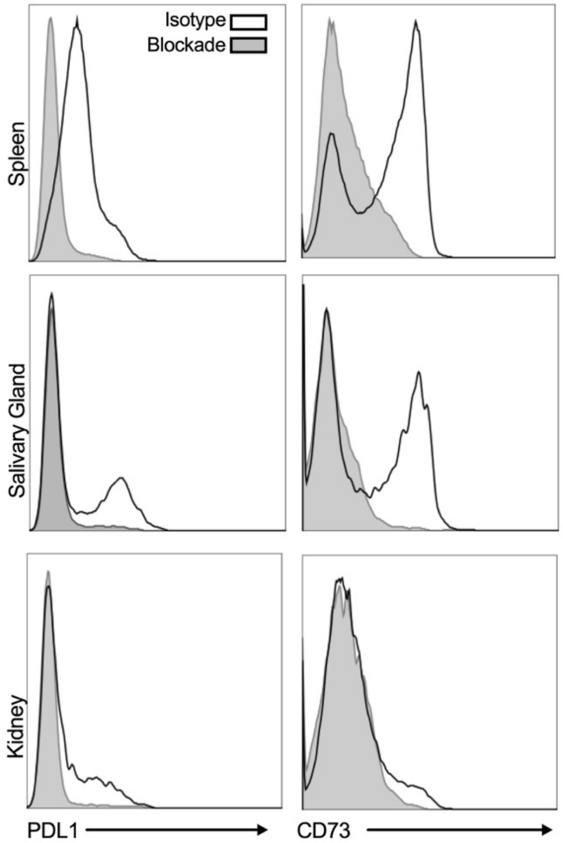

Figure S6: *In vivo* antibody blockade saturates target receptors in non-lymphoid organs
Mice were seeded with OT-Is and infected with MCMV-OVA for at least 3 months and then treated with isotype control antibodies, or anti-PD-L1 (left, clone 10F.9.G2) or anti-CD73 (right, clone TY/23) for 2 weeks before sacrifice, as described in the methods. Upon sacrifice, cells recovered from the indicated organs were stained with fluorescently-labeled versions of the same clones used for blockade. Data are representative for n=6 mice from 2 independent experiments.

Supplemental Figure 7

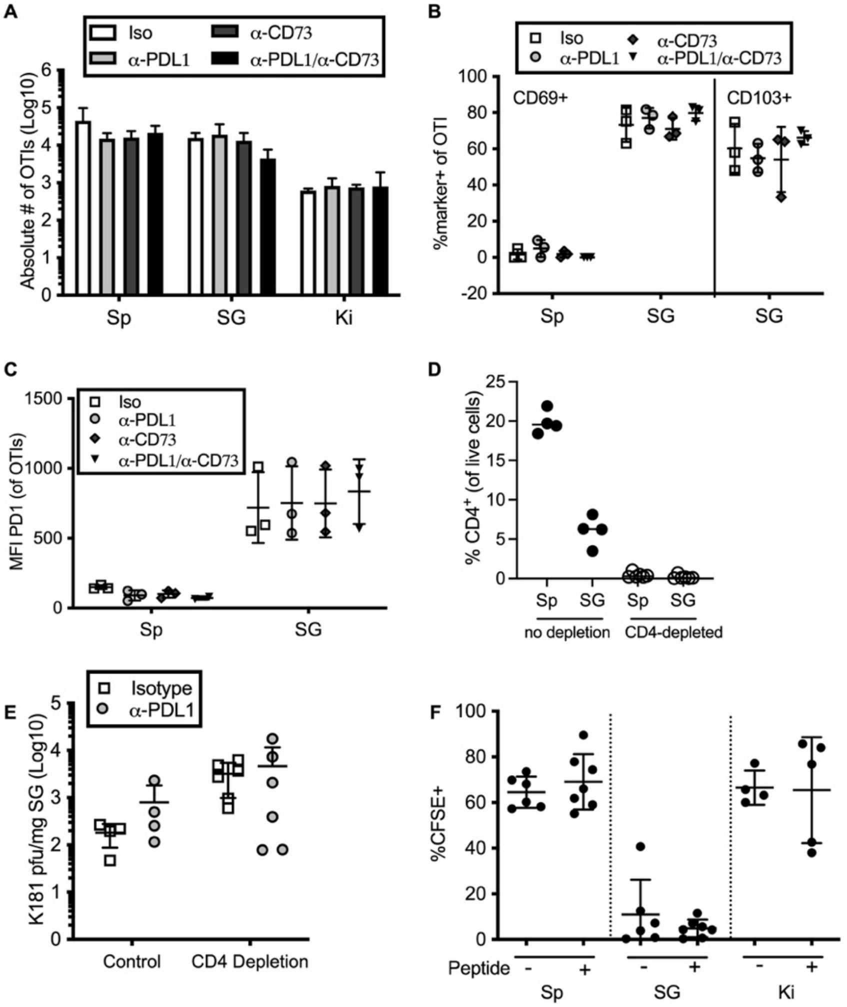

Figure S7: ***In vivo*** **effects of inhibitory antibody blockade.**
Mice were seeded with OT-Is and infected with MCMV-OVA and then treated with the indicated blocking antibodies or isotype control antibodies between day 7 and day 21 of infection. Mice were sacrificed on day 21 post infection. Fluorochrome labelled CD8α antibody was used to distinguish between vascular and parenchymal T cells as described in the methods. A) Absolute numbers of OT-Is in the spleen (Sp), salivary gland (SG), or Kidney (Ki) after antibody blockade. B) Frequency of expression of CD69 (left) or CD103 (right) on OT-Is in the spleen or salivary gland after antibody blockade. C) Mean fluorescence intensity of PD1 on OT-Is in the spleen or salivary gland after antibody blockade. D) Efficacy of CD4 T cell depletion using antibody (clone GK1.5) on day -2, -1 and 0 of infection and then weekly thereafter. CD4 T cell numbers were assessed with clone RM4.4. E) Mice with or without CD4 T cell depletion were infected with wild type MCMV (strain K181) and treated with anti-PD-L1 blocking antibody starting at day 7 post infection until sacrifice at 1 month post infection. Salivary glands were harvested and homogenized for plaque assays. Shown are the plaque forming units of MCMV from the salivary gland. Data are pooled from n=4-6 mice from two independent experiments. F) Mice were seeded with OT-Is and infected with MCMV-OVA for at least 3 months. Mice were injected i.v. with 200 μg of SIINFEKL peptide and CFSE as previously described (Smith et al., 2015) and sacrificed 6 hours later. Shown is the frequency of $CFSE^+$ OT-Is in the indicated organs. Data are pooled from n = 6 mice, from 2 independent experiments.

Supplemental Figure 8

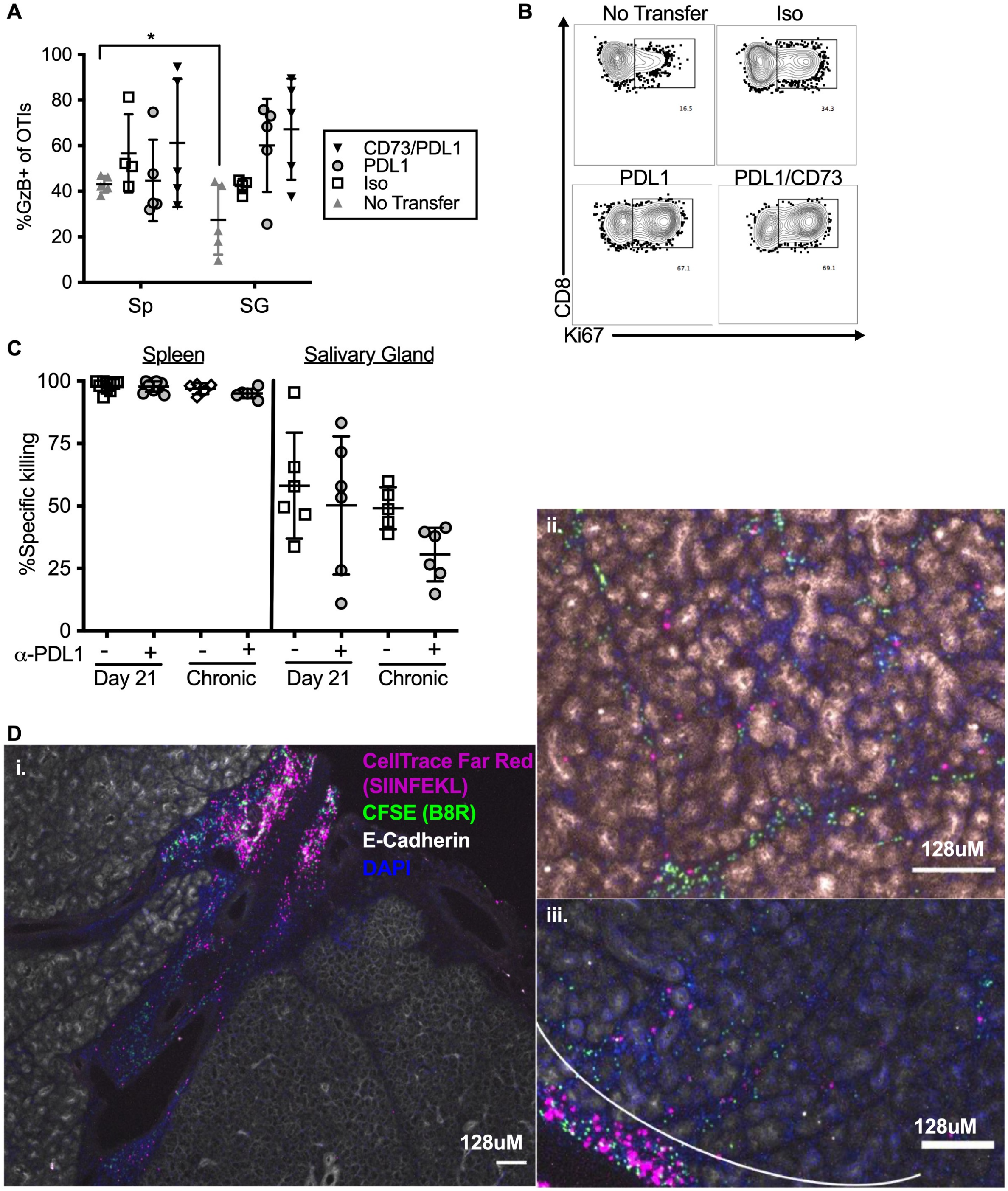

Figure S8: ***In vivo*** **killing assay**

Mice were seeded with OT-I T cells and infected with MCMV-Ova for 21 days (A-C) or more than 12 weeks (C, chronic). For injection of target cells, splenocytes from naïve mice were pulsed with either SIINFEKL peptide or an irrelevant peptide (B8R) and labelled with CellTrace Far Red or CFSE respectively. Target cells were injected i.v. and intra-glandularly (i.g.) into OT-I bearing mice that had been treated with the indicated blocking antibodies or isotype control antibodies for the two weeks prior to injection of target cells. Upon sacrifice, half of the harvested organs were used to harvest lymphocytes

for FACS analysis and half were frozen in OCT to analyze target cell killing by histology. A) Frequency of granzyme B expression among OT-Is in the salivary gland. B) Representative FACS plots of Ki-67 expression by OT-Is in the salivary gland. Data are from n=5 mice, from 2 independent experiments. C) Specific killing of target cells as assessed by flow cytometry from killing assays done at day 21 or chronic time points (>12 weeks) post infection. Data are from n=6 mice per group, from 2 independent experiments. D) Representative images of target cells in the salivary gland 2 days after injection. Image (i.) shows a low magnification image of cells stuck between lobules. Images (ii.) and (iii.) show higher magnification images of E-cadherin^{+} areas. Curved white line on image (iii.) shows representative gating strategy for cells in the E-cadherin+ region of the salivary gland.
